# Strategies for prevention of postoperative delirium: a systematic review and meta-analysis of randomized trials

**DOI:** 10.1186/cc12566

**Published:** 2013-03-18

**Authors:** Hao Zhang, Yan Lu, Meng Liu, Zui Zou, Long Wang, Feng-Ying Xu, Xue-Yin Shi

**Affiliations:** 1 Department of Anesthesiology and Neuroscience Research Center, Changzheng Hospital, Second Military Medical University, 415 Fengyang Road, Shanghai 200003, China; 2 Institute of Neuroscience and MOE Key Laboratory of Molecular Neurobiology, Second Military Medical University, 800 Xiangyin Road, Shanghai 200433, China; 3 Department of Anesthesiology, Eastern Hepatobiliary Hospital, 225 Changhai Road, Shanghai 200438, China

## Abstract

**Introduction:**

The ideal measures to prevent postoperative delirium remain unestablished. We conducted this systematic review and meta-analysis to clarify the significance of potential interventions.

**Methods:**

The PRISMA statement guidelines were followed. Two researchers searched MEDLINE, EMBASE, CINAHL and the Cochrane Library for articles published in English before August 2012. Additional sources included reference lists from reviews and related articles from 'Google Scholar'. Randomized clinical trials (RCTs) on interventions seeking to prevent postoperative delirium in adult patients were included. Data extraction and methodological quality assessment were performed using predefined data fields and scoring system. Meta-analysis was accomplished for studies that used similar strategies. The primary outcome measure was the incidence of postoperative delirium. We further tested whether interventions effective in preventing postoperative delirium shortened the length of hospital stay.

**Results:**

We identified 38 RCTs with interventions ranging from perioperative managements to pharmacological, psychological or multicomponent interventions. Meta-analysis showed dexmedetomidine sedation was associated with less delirium compared to sedation produced by other drugs (two RCTs with 415 patients, pooled risk ratio (RR) = 0.39; 95% confidence interval (CI) = 0.16 to 0.95). Both typical (three RCTs with 965 patients, RR = 0.71; 95% CI = 0.54 to 0.93) and atypical antipsychotics (three RCTs with 627 patients, RR = 0.36; 95% CI = 0.26 to 0.50) decreased delirium occurrence when compared to placebos. Multicomponent interventions (two RCTs with 325 patients, RR = 0.71; 95% CI = 0.58 to 0.86) were effective in preventing delirium. No difference in the incidences of delirium was found between: neuraxial and general anesthesia (four RCTs with 511 patients, RR = 0.99; 95% CI = 0.65 to 1.50); epidural and intravenous analgesia (three RCTs with 167 patients, RR = 0.93; 95% CI = 0.61 to 1.43) or acetylcholinesterase inhibitors and placebo (four RCTs with 242 patients, RR = 0.95; 95% CI = 0.63 to 1.44). Effective prevention of postoperative delirium did not shorten the length of hospital stay (10 RCTs with 1,636 patients, pooled SMD (standard mean difference) = -0.06; 95% CI = -0.16 to 0.04).

**Conclusions:**

The included studies showed great inconsistencies in definition, incidence, severity and duration of postoperative delirium. Meta-analysis supported dexmedetomidine sedation, multicomponent interventions and antipsychotics were useful in preventing postoperative delirium.

## Introduction

An estimated 36.8% of surgical patients suffer from postoperative delirium [1]. The incidence is much higher in patients 70 years of age and older [2]. Delirium is associated with increased morbidity and mortality [3], prolonged hospital stay and persistent functional and cognitive decline [4]. Postoperative delirium is also a major burden to medical services with costs in US dollars ranging from $38 to $152 billion per year [5].

Prevention may be the most effective strategy for minimizing the occurrence of postoperative delirium and its adverse outcomes but it is untested or unproven. In hospitalized patients, 30 to 40% cases of delirium are thought to be preventable [6,7]. Multimodal strategies have been used in an effort to counter delirium resulting from diverse causes such as neurotransmitter imbalance, neuroinflammation, pain, infection, metabolic abnormalities and sleep disorders [8,9]. Widely applicable therapeutic countermeasures for delirium have not yet been discovered. It is not presently clear whether a single intervention for patients with different risk factors is a realistic goal, or whether there is an optimal treatment for specific groups of patients.

The purposes of this study were 1) to critically review available randomized clinical trials (RCTs) that assessed the effects of multiple kinds of interventions to prevent postoperative delirium in adult patients, 2) to determine the efficacy of interventions, and 3) to explore whether interventions successful in preventing postoperative delirium also shortened the length of hospital stay.

## Materials and methods

This systematic review and meta-analysis was conducted following the guidelines of the PRISMA statement (Additional file 1) [10,11].

### Search strategy

We conducted a literature search of MEDLINE, EMBASE, CINAHL and the Cochrane Library databases for articles published in English before August, 2012. Search key words were delirium (including delirium, confusion, acute confusional state or acute confusional syndrome) and postoperative (including postoperative, operation, surgery, anaesthesia or anesthesia). We only searched articles reporting results from adult patients. Case reports were excluded from our primary search. The search strategy we used for MEDLINE was as follows: 1) *delirium*; 2) deliri*; 3) *confusion*; 4) *acute confusional state*; 5) *acute confusional syndrome*; 6) *postoperative*; 7) operation*; 8) *surgery*; 9) *surgical*; 10) *anaesthesia*; 11) *anesthesia*; 12) *1 OR 2 OR 3 OR 4 OR 5*; 13) *6 OR 7 OR 8 OR 9 OR 10 OR 11*; 14) *12 OR 13*; 15) '*English*' (*Language*); 16) *14 AND 15*; 17) '*case reports*' (*Publication Type)*; 18) *16 NOT 17*; 19) *'Adult' (Mesh)*; 20) *18 AND 19*. Additional studies were identified by reviewing the reference lists of reviews and meta-analyses and searching the related articles of identified studies using 'Google Scholar'.

### Study selection

The initial search returned 2,813 articles. After title and abstract review, 198 potential articles with full texts were further independently reviewed by two coauthors (HZ and YL) to determine the eligibility according to the predefined selection and exclusion criteria. Disagreements between reviewers were resolved by including another coauthor (XS). Completed studies that met all the following criteria were considered eligible for inclusion in the systematic review and meta-analysis: 1) RCTs assessing interventions to prevent postoperative delirium; 2) delirium identified by validated methods including the Diagnostic and Statistical Manual of Mental Disorders, 1987 (DSM-III), DSM-III-R (1994), DSM-IV (1999), the 10th revision of the International Statistical Classification of Diseases and Related Health Problems, 1992 (ICD-10), and clinical diagnostic tools based on these such as the Confusion Assessment Method (CAM), Delirium Rating Scale (DRS) and NEECHAM Confusion Scale [12]; 3) incidence, severity and duration of delirium analyzed independently of other neurologic events such as emergence delirium and dementia. Research articles were excluded if they recruited 1) patients with delirium before surgery; 2) patients with alcohol withdrawal syndrome; 3) groups that also included nonsurgical patients (for example patients in the intensive care unit or ward without surgery); 4) homogeneous populations of patients with certain central nervous system diseases or mental disorders (for example stroke, dementia, schizophrenia and depression).

### Data extraction

Data extraction was completed by two coauthors (ML and ZZ) using a predesigned piloted data extraction form. Disagreements were resolved by the third coauthor (XS) consultation. The following study characteristics were collected: primary author, publication year, country of origin, PubMed identifier (if possible), types of surgery, participant characteristics (gender, age, number, existing illness, inclusion and exclusion criteria), intervention (type, dosage, duration and frequency), criteria for delirium, incidence, severity and duration of delirium, *P *value, duration and frequency of follow-up and the length of hospital stay. Dichotomous data were converted to incidences for data synthesis and continuous data were recorded using mean and standard deviation (SD).

### Quality scoring of included trials

The validity and quality of included trials was evaluated independently by two coauthors (FX and LW) using a scoring system (Table 1) that combined the modified Jadad scale [13] and the delirium-specific score we developed for the current study. The quality review system included eight items with a maximal score of 12. Studies with a score ≤ 5 were arbitrarily defined as low-quality studies with high risk of within-study bias. We designed this delirium-specific scoring system because postoperative delirium is defined subjectively with validated methods such as DSM-IV and ICD-10, has certain risk factors (for example age, sex, comorbidities and medications) and mostly occurs 24 to 72 hours after surgery [1,12,14-16]. Disagreements were resolved by including a third author (XS) for discussion. Studies were not excluded or weighted based on quality scores in the meta-analysis.

**Table 1:** The quality review system for included trials.

	Item	Score	Criteria
		2	Randomization is described and adequate (random numbers, computer generated, etc.)
	**Randomization**	1	Randomization is described
		0	No or inappropriate randomization
	
	**Allocation**	2	Allocation concealment is described and adequate (sequentially numbered opaque sealed envelopes, central randomization, etc.)
	**concealment**	1	Allocation concealment is described
**Adapted Jadad score**		0	No or inappropriate allocation concealment
	
	**Intervention**	2	Blinding is described and adequate
	**blinding**	1	Blinding is described
		0	No or inappropriate blinding
	
	**Withdrawal or**	1	Withdrawals and dropouts are described
	**dropouts**	0	Withdrawals or dropouts are not described
	
	**Intention-to-treat**	1	ITT analysis is used
	**(ITT) analysis**	0	ITT analysis is not used

	**Similar groups at baseline**	1	Delirium-related factors (age, sex, pre-existing cognitive or sensory deficit, physical functional status, comorbid diseases, medications and alcohol consumption [12,15,16]) are similar between groups
		0	Delirium-related factors are not screened or different
	
**Delirium specific score**	**Delirium assessor blinding**	1	Delirium assessor is blinded to the interventions
			Delirium assessor is not blinded to the interventions
	
		2	Frequency: ≥ 1/day since postoperative day (POD) 1 and duration: > 3 days since POD 1 [1,14]
	**Delirium follow-up**	1	Frequency: ≥ 1/day since POD 1 and duration: POD 1-3
		0	Frequency: < 1/day or duration < 3 days since POD 1

### Data analysis

The analyses focused on the incidence of postoperative delirium as the primary outcome measure. We further tested the hypothesis that interventions reducing postoperative delirium would shorten the length of hospital stay. Only studies reporting significant differences in the incidences of postoperative delirium between two interventions (*P *< 0.05, two-tailed) were included. Placebo and control procedures were also considered as interventions when a study aimed to compare the effects between interventions and placebos. Interventions were divided into two groups (interventions with less delirium and interventions with more delirium) and the length of hospital stay was synthesized for comparison.

Meta-analysis was performed when two or more than two studies using similar interventions were identified. Statistical analysis was performed using STATA 11 (StataCorp, College Station, TX, USA). A test for heterogeneity was performed using a standard chi-square (χ^2^) and I-square (I^2^) statistic. Significant heterogeneity was considered present at χ^2 ^*P *< 0.10 or I^2 ^> 50%. Where no heterogeneity was found, a fixed-effects parametric approach (weighted with inverse variance) was taken. Otherwise a random-effects model was used. For the incidence of postoperative delirium, both pooled relative risk (RR) and incidence with 95% confidence intervals (CI) were calculated. Sample size calculations of different interventions (n1 = n2, α = 0.05 and β = 0.1, two-tailed) were performed based on reported or pooled incidences. For the length of hospital stay, SMD (standard mean difference) was used due to that there was a big intertrial difference. We intended to conduct a subgroup analysis, where possible, to explore 1) the effects of different interventions, or 2) the effects of single intervention in patients with different surgeries. Publication bias was assessed by visually inspecting funnel plot and Begg's test. Meta-regression was performed to help investigate the origin of heterogeneity. For all the analyses, a *P *value of less than 0.05 (two-tailed) was considered statistically significant.

## Results

### Study selection

**Figure 1:**
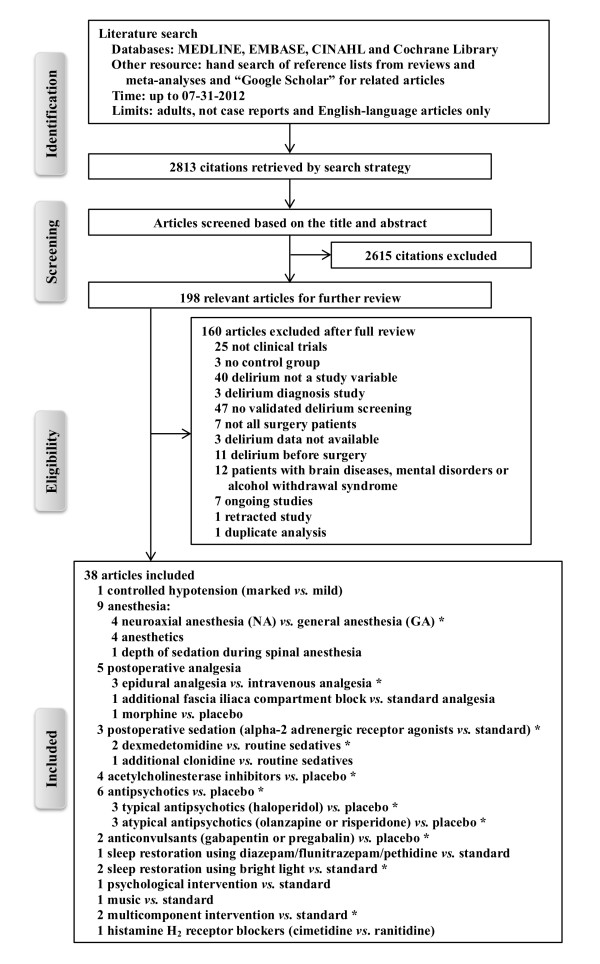
**Flow chart of identification, screening, review and selection of studies**. *indicates the group of studies identified for meta-analysis.

The process of literature identification, screening and selection is summarized by Figure 1. Our primary search yielded 2,813 articles. After screening, 198 studies potentially met the inclusion criteria. After examining the full texts, 160 articles were excluded: 25 studies were not clinical trials; three studies had no control group; 40 studies did not include postoperative delirium as a study variable; three studies tested the diagnosis methods of delirium; 47 studies did not screen postoperative delirium using validated tools; seven studies recruited both surgical and nonsurgical patients; three studies did not provide the delirium data; 11 studies included patients with delirium prior to surgery; 12 studies included homogeneous patients with brain diseases, mental disorders or alcohol withdrawal syndrome; seven studies described ongoing trials; one study was retracted, and one study was identified as a duplicated publication. We ultimately included 38 RCTs [17-54] in our systematic review and meta-analysis.

### Study characteristics

The characteristics of 38 included studies were listed in Table 2 and Table 3. These single-centered studies [17-54] included from 11 [38] to 457 [54] patients. The earliest study was published in 1987 [17]. The average ages of the participants were all above 60 except two studies by Leung [33] and Maldonado [41]. Two studies included patients only at high risk for delirium [29,43]. One study included patients only with subsyndromal delirium [52]. The surgery types included orthopedic (*n *= 18) [17,19,20,23,25,27,29,30,33,35,37,39,42,43,45,47,48,53], cardiovascular (*n *= 9) [18,21,36,40,41,44,46,51,52], abdominal (*n *= 7) [22,24,26,28,31,32,50], noncardiovascular (*n *= 2) [34,54], and thoracic (*n *= 2) surgeries [38,49]. Interventions could be divided into two categories. Category 1, perioperative procedures and drugs (*n *= 18, Table 2) including controlled hypotension [23], anesthesia [17,20,27,28,31,34,40,47,51], analgesia [19,24,32,43,53] and sedation [41,44,46]. Category 2, pharmacological, psychological or multicomponent interventions (*n *= 20, Table 3) including acetylcholinesterase inhibitors [30,37,39,48], antipsychotics [22,29,36,45,52,54], anticonvulsants [33,50], sleep restoration by drug [26] or bright light [38,49], psychological intervention [18], music [42], multicomponent interventions [25,35] and histamine H_2 _receptor blockers [21]. Thirty-six studies [17-41,43-49,51-54] reported incidences of postoperative delirium. The reported incidences ranged from 0 [18,28,33] to 75.3% [35]. Inpatient time was reported in 22 studies [18,20,21,24-26,29,30,32,34-37,39-41,44,47,49,51,52,54]. The duration of postoperative delirium was reported in 10 studies [25,29,30,35,41,43-45,47,52] and the severity of delirium was reported in 11 studies [25,28,29,38,42,43,45,46,48,50,53].

**Table 2:** Characteristics of included studies evaluating different perioperative procedures and drugs

Subcategory	Study	Definition delirium	Evaluation timing (d)	Surgery type	Intervention (I1 *vs. *I2)	Gender (F/M)	Age (y)	Score	Incidence (n/total) (%)	*P *value	Delirium duration or severity	Hospital stay (d)
**Controlled hypotension**	Williams-Russo, 1999 [23]	DSM- III	POD 1-7 or discharge	Orthopedic	MAP 45-55 *vs. *MAP 55-70 mmHg	I1: 51/66 I2: 65/53	I1: 72 ± 7 I2: 72 ± 8	10	I1: 10/117 (8.5%) I2: 5/118 (4.2%)	0.177		N/A

	Berggren, 1987 [17]	DSM- III	POD 1,7	Orthopedic	Neuraxial (epidural) *vs. *general anesthesia (halothane)	I1: 22/7 I2: 24/4	I1: 78 ± 8 I2: 77 ± 7	5	I1: 11/26 (42.3%) I2: 9/26 (34.6%)	0.569		N/A, NS
**Anesthesia type (neuraxial or general anesthesia)**	Williams-Russo, 1995 [20]	DSM- III	POD 1-7	Orthopedic	Neuraxial (epidural) *vs. *general anesthesia (isoflurane)	I1: 71/63 I2: 70/58	I1: 69 I2: 69	9	I1: 16/134 (11.9%) I2: 12/128 (9.4%)	0.502		I1: 12.7 ± 5.3 I2: 12.7 ± 4.3
	Kudoh, 2004 [27]	CAM	POD 1-7	Orthopedic	Neuraxial (epidural) *vs. *general anesthesia (propofol)	I1: 69/6 I2: 66/9	I1: 76 ± 4 I2: 75 ± 4	8	I1: 5/75 (6.7%) I2: 12/75 (16%)	0.070		N/A
	Papaioannou, 2005 [31]	DSM-III	POD 1-3	Abdominal	Neuraxial (some patients used propofol for sedation) *vs. *general anesthesia	I1: 10/18 I2: 7/12	> 60	7	I1: 6/28 (21.4%) I2: 3/19 (15.8%)	0.720		N/A

**Sedation depth during spinal anesthesia**	Sieber, 2010 [47]	CAM	POD 1-discharge	Orthopedic	Deep sedation (BIS ≈ 50) *vs. *light sedation (BIS ≥ 80)	I1: 43/14 I2: 40/17	I1: 82 ± 7 I2: 81 ± 8	9	I1: 23/57 (40.4%) I2: 11/57 (19.3%)	**0.014**	**Duration (*P *= 0.01):** I1: 1.4 ± 4.0 days I2: 0.5 ± 1.5 days **Duration in patients with delirium (*P *= 0.77):** I1: 3.4 ± 5.7 days I2: 2.8 ± 2.3 days	I1: 4.5 ± 2.3 I2: 4.7 ± 3.1

	Nishikawa, 2004 [28]	DSM-III, DRS	POD 1-3	Abdominal	Epidural anesthesia/propofol *vs. *epidural anesthesia/sevoflurane	I1: 12/13 I2: 13/12	I1: 71 ± 8 I2: 71 ± 7	7	I1: 4/25 (16%) I2: 0/25 (0)	0.110	**Severity based on DRS (*P *= 0.002):** I1: 6 ± 3 I2: 2 ± 1	N/A
**Anesthesia (anesthetics)**	Hudetz, 2009 [40]	DSM-IV	POD 1-5	Cardiovascular	Additional ketamine (0.5 mg/kg, iv, single bolus) *vs. *standard	N/A	I1: 68 ± 8 I2: 60 ± 8	8	I1: 1/29 (3.4%) I2: 9/29 (31.0%)	**0.012**		I1: 8 ± 4 I2: 7 ± 3
	Royse, 2011 [51]	CAM	POD 1	Cardiovascular	propofol *vs. *desflurane	I1: 9/80 I2: 18/73	I1: 64 ± 11 I2: 62 ± 10	8	I1: 7/89 (7.9%) I2: 12/92 (13.0%)	0.245		I1: 7 ± 2 I2: 6 ± 2
	Leung, 2006 [34]	CAM	POD 1-2	Non-cardiovascular	Additional N_2_O *vs. *standard	I1: 62/52 I2: 51/63	I1: 74 ± 6 I2: 74 ± 6	6	I1: 44/105 (41.9%) I2: 46/105 (43.8%)	0.780		I1: 5.4 ± 3.5 I2: 4.8 ± 2.9

**Postoperative analgesia (epidural or intravenous analgesia)**	Williams-Russo, 1992 [19]	DSM- III	POD 1-7	Orthopedic	Epidural analgesia *vs. *Intravenous analgesia	N/A	68 ± 7	7	I1: 10/26 (38.5%) I2: 11/25 (44%)	0.688		N/A
	Mann, 2000 [24]	DSM-III	POD 1- discharge	Abdominal	Epidural analgesia *vs. *Intravenous analgesia	I1: 15/20 I2: 17/18	I1: 76 ± 6 I2: 77 ± 5	8	I1: 8/31 (25.8%) I2: 8/33 (24.2%)	0.885		I1: 10.5 ± 5 I2: 11.5 ± 6
	Beaussier, 2006 [32]	CAM	POD 1- discharge	Abdominal	Epidural analgesia *vs. *Intravenous analgesia	I1: 11/15 I2: 14/12	I1: 78 ± 5 I2: 77 ± 5	9	I1: 9/26 (34.6%) I2: 10/26 (38.5%)	0.773		I1: 7.9 ± 2 I2: 8.4 ± 1.7

**Postoperative analgesia (additional regional analgesia or standard treatment)**	Mouzopoulos, 2009 [43]	DSM-IV, CAM, DRS	POD 1- discharge	Orthopedic	Additional fascia iliaca compartment block (0.25% bupivacaine, 0.3 mL/kg) *vs. *standard treatment	I1: 78/24 I2: 76/29	I1: 72 ± 4 I2: 73 ± 4	7	I1: 11/102 (10.8%) I2: 25/105 (23.8%)	**< 0.001**	**Duration (*P *< 0.001):** I1: 5.22 ± 4.28 days I2: 10.97 ± 7.16 days **Severity based on DRS (*P *< 0.001):** I1: 14.34 ± 3.6 I2: 18.61 ± 3.4	N/A

**Postoperative analgesia (morphine or placebo)**	Musclow, 2012 [53]	NEECHAM	POD 1- discharge	Orthopedic	Morphine (30 mg, bid, po) (POD1-3) *vs. *placebo	I1: 78/24 I2: 76/29	I1: 67 ± 4 I2: 64 ± 11	11	I1: 10/97 (10.3%) I2: 3/93 (3.2%)	0.082	**Severity based on NEECHAM (*P *= 0.02):** I1: 28.70 ± 1.82 I2: 29.14 ± 0.61	N/A

**Postoperative sedation (alpha-2 adrenergic receptor agonists or other sedatives)**	Maldonado, 2009 [41]	DSM-IV	POD 1-3	Cardiovascular	Dexmedetomidine (loading dose: 0.4 μg/kg, maintenance drip of 0.2-0.7 μg/kg/hour) *vs. *propofol (25-75 μg/kg/min) *vs. *midazolam (0.5-2 mg/hour)	I1: 14/26 I2: 16/22 I3: 13/27	I1: 55 ± 16 I2: 58 ± 18 I3: 60 ± 16	8	I1: 4/40 (10%) I2: 16/36 (44.4%) I3: 17/40 (42.5%)	**< 0.001**	**Duration in patients with delirium (*P *= 0.82):** I1: 2.0 ± 0 days I2: 3.0 ± 3.1 days I3: 5.4 ± 6.6 days	I1: 7.1 ± 1.9 I2: 8.2 ± 3.8 I3: 8.9 ± 4.7
	Shehabi, 2009 [44]	CAM-ICU	POD 1-5	Cardiovascular	Dexmedetomidine (0.1-0.7 μg/kg/hour) *vs. *morphine (10-70 μg/kg/hour)	I1: 38/114 I2: 36/111	I1: 72 ± 8 I2: 71 ± 8	11	I1: 13/152 (8.6%) I2: 22/147 (15.0%)	**0.031**	**Duration (*P *= 0.032):** I1: 2 ± 4 days I2: 5 ± 8 days	I1: 8 ± 3 I2: 8 ± 3
	Rubino, 2010 [46]	DSM-IV, DDS	30 minutes after weaning	Cardiovascular	Additional clonidine (loading dose: 0.5 μg/kg, maintenance drip of 1-2 μg/kg/hour) *vs. *standard	I2: 5/10 I2: 7/8	I1: 64 ± 9 I2: 61 ± 6	8	I1: 6/15 (40%) I2: 5/15 (30%)	0.705	**Severity based on DDS (*P *< 0.001):** I1: 0.6 ± 0.7 I2: 1.8 ± 0.8	N/A

N/A, not available; NS, not significant; POD, postoperative day; DDS, Delirium Detection Score; DSM, Diagnostic and Statistical Manual of Mental Disorders; CAM, Confusion Assessment Method; DRS, Delirium Rating Scale.

**Table 3:** Characteristics of included studies evaluating pharmacological, psychological or multicomponent interventions

Subcategory	Study	Definition delirium	Evaluation timing (d)	Surgery type	Intervention (I1 *vs. *I2)	Gender (F/M)	Age (y)	Score	Incidence (n/total) (%)	*P *value	Delirium duration or severity	Hospital stay (d)
	Liptzin, 2005 [30]	DSM-IV, CAM, DSI	POD 7, POD 14	Orthopedic	Donepezil (5 mg, po) (pre-1-14+ POD 1-14) *vs. *placebo	I1: 25/14 I2: 21/20	I1: 67 ± 9 I2: 68 ± 9	6	I1: 8/39 (20.5%) I2: 7/41 (17.1%)	0.694	**Duration (*P *= 0.12):** I1: 1 ± 0 days I2: 1.3 ± 1.2 days	I1: 4.4 ± 0.8 I2: 4.2 ± 0.5
	Sampson, 2007 [37]	DSI	POD 1-4	Orthopedic	Donepezil (5 mg, po) (pre- + POD 1-3) *vs. *placebo	I1: 8/11 I2: 6/8	I1: 70 ± 8 I2: 65 ± 11	10	I1: 2/19 (10.5%) I2: 5/14 (35.7%)	0.106		I1: 9.9 ± 3.2 I2: 12.1 ± 4.1
**Cholinesterase inhibitors**	Marcantonio, 2011 [48]	CAM, DSI, MDAS	POD 1-discharge; 2, 4, and 6 weeks	Orthopedic	Donepezil (5 mg, po) (POD 1-30) *vs. *placebo	I1: 5/2 I2: 4/5	I1: 88 ± 5 I2: 87 ± 4	9	I1: 3/7 (42.9%) I2: 4/9 (44.4%)	1	**Severity based on MDAS changes (*P *= 0.91):** I1: 1.3 ± 2.5 I2: 1.6 ± 5.2	N/A
	Gamberini, 2009 [39]	CAM	POD 1-6	Orthopedic	Rivastigmine (1.5 mg, tid, po) (pre-1 + POD 1-6) *vs. *placebo	I1: 19/37 I2: 17/40	I1: 74 ± 5 I2: 74 ± 6	10	I1: 18/56 (32.1%) I2: 17/57 (29.8%)	0.790		I1: 13 ± 6.2 I1: 13 ± 6.2

	Kaneko, 1999 [22]	DSM-III-R	POD 5	Abdominal	Haloperidol (5 mg, iv) (POD 1-5) *vs. *saline	I1: 14/24 I2: 14/26	I1: 72 ± 8 I2: 73 ± 9	5	I1: 4/38 (10.5%) I2: 13/40 (32.5%)	**0.027**		N/A
**Antipsychotics (typical)**	Kalisvaart, 2005 [29]	DSM-IV, CAM, DRS	POD 1-3	Orthopedic	Haloperidol (0.5 mg, tid, po) (pre-POD 3) *vs. *placebo	I1: 172/40 I2: 171/47	I1: 79 ± 6 I2: 80 ± 6	11	I1: 32/212 (15.1%) I2: 36/218 (13.8%)	0.687	**Severity in patients with delirium based on DRS (*P *< 0.001):** I1: 14.40 ± 3.5 I2: 18.41 ± 4.4 **Duration in patients with delirium (*P *< 0.001):** I1: 5.41 ± 4.91 days I2: 11.85 ± 7.56 days	Patients with delirium: I1: 17.1 ± 11.1 I2: 22.6 ± 16.7
	Wang, 2012 [54]	CAM-ICU	POD 1-7	Non-cardiovascular	Haloperidol (1.7 mg, iv) (POD) *vs. *saline	I1: 84/145 I2: 85/143	I1: 74 ± 6 I2: 74 ± 7	12	I1: 35/229 (15.3%) I2: 53/228 (23.2%)	0.031		I1: 11.0 ± 0.9 I1: 11.0 ± 0.8

	Larsen, 2010 [45]	DSM-III-R, CAM, DRS	POD 1-8 or discharge	Orthopedic	Olanzapine (5 mg, po) (pre- + POD) *vs. *placebo	I1: 94/102 I2: 123/81	I1: 73 ± 6 I2: 74 ± 6	9	I1: 28/196 (14.3%) I2: 82/204 (40.2%)	< 0.001	**Severity based on DRS (*P = *0.02):** I1: 16.44 ± 3.7 I2: 14.5 ± 2.7 **Duration (*P *= 0.02):** I1: 2.2 ± 1.3 days I2: 1.6 ± 0.7 days	N/A
**Antipsychotics (atypical)**	Prakanrattana, 2007 [36]	CAM-ICU	POD 1-discharge	Cardiovascular	Risperidone (1 mg, sl) (POD) *vs. *placebo	I1: 27/36 I2: 25/38	I1: 61 ± 10 I2: 61 ± 10	12	I1: 7/63 (11.1%) I2: 20/63 (31.7%)	0.009		I1: 10.5 ± 6.1 I2: 10.3 ± 4.4
	Hakim, 2012 [52]	DSM-IV	ICU -discharge	Cardiovascular	Risperidone (0.5 mg, bid, po) (POD until 24 hours after subsidence of subsyndromal delirium or a score of more than 3 on the ICDSC was obtained) *vs. *placebo	I1: 18/33 I2: 14/36	> 65	12	I1: 7/51 (13.7%) I2: 17/50 (34%)	0.031	**Duration in patients with delirium (*P *= 0.669):** I1: 3 ± 1.5 days I2: 3 ± 0.8 days	I1: 6 ± 1.5 I2: 6 ± 2.3

	Leung, 2006 [33]	CAM	POD 1-3	Orthopedic	Gabapentin (900 mg, po) (pre- + POD 1-3) *vs. *placebo	I1: 5/4 I2: 5/7	I1: 57 ± 10 I2: 61 ± 11	10	I1: 0/9 (0) I2: 5/12 (41.7%)	0.045		N/A
**Anticonvulsants**	Pesonen, 2011 [50]	CAM-ICU	POD 1-5	Abdominal	Pregabalin (150 mg, po) (pre- + POD 1-5) *vs. *placebo	I1: 14/21 I2: 19/16	I1: 80 ± 11 I2: 80 ± 12	10	N/A	N/A	**Severity based on CAM-ICU (*P *= 0.04):** I1: 24 ± 8 I2: 21 ± 19	N/A

**Sleep restoration (diazepam/flunitrazepam/pethidine)**	Aizawa, 2002 [26]	DSM-IV	POD 1-7	Abdominal	Diazepam (0.1 mg/kg, im)/flunitrazepam (0.04 mg/kg, iv)/pethidine (1 mg/kg, iv) (POD 1-3) *vs. *standard	I1: 5/15 I2: 9/11	I1: 76 ± 5 I2: 76 ± 4	8	I1: 1/20 (5%) I2: 7/20 (35%)	0.023		I1: 25.6 ± 9.4 I2: 29.9 ± 6.2

**Sleep restoration (Bright light)**	Taguchi, 2007 [38]	NEECHAM	POD 1-5	Thoracic	Bright light (2 hours per day; morning; 5000 lx) *vs. *standard	I1: 0/6 I2: 0/5	I1: 56 ± 14 I2: 59 ± 14	7	I1: 1/6 (16.7%) I2: 2/5 (40%)	0.545	**Severity based on NEECHAM (*P *= 0.014):** I1: 6.7 ± 0.7 I2: 21.1 ± 7	N/A
	Ono, 2011 [49]	NEECHAM, DSM-IV	POD 1-6	Thoracic	Bright light (POD2-5; 2 hours per day; morning; 2500-5000 lx) *vs. *standard	I1: 0/6 I2: 0/5	I1: 63 ± 10 I2: 64 ± 8	6	I1: 1/10 (10%) I2: 5/12 (41.7%)	0.162		I1: 24.8 ± 3.9 I2: 24.8 ± 4.0

**Psychological interventions**	Schindler, 1989 [18]	DSM-III	discharge	Cardiovascular	Daily psychiatric intervention *vs. *standard	I1: 13/3 I2: 13/4	I1: 58 ± 8 I1: 61 ± 6	3	I1: 0/17 (0) I2: 2/16 (12.5%)	0.227		I1: 15.7 ± 5 I2: 18.7 ± 6

**Music**	McCaffrey, 2009 [42]	NEECHAM	POD 1-3	Orthopedic	Music *vs. *standard	I1: 7/4 I2: 7/4	I1: 75 ± 5 I1: 76 ± 6	6	N/A	N/A	**Severity based on NEECHAM (*P *= 0.000):** I1: 24 ± 0.97 I2: 22.5 ± 1.22	N/A

**Multi-component interventions**	Marcantonio, 2001 [25]	CAM	POD 1-discharge	Orthopedic	Geriatrics consultation (a geriatrician made daily visits for the duration of the hospitalization and made targeted recommendations based on a structured protocol including 10 modules) *vs. *standard	I1: 49/13 I2: 50/14	I1: 78 ± 8 I2: 80 ± 8	9	I1: 20/62 (32.3%) I2: 32/64 (50%)	0.043	**The number of patients with severe delirium (*P *= 0.02):** I1: 7/62 (11.3%) I2: 18/64 (28.1%) **Duration in patients with delirium (*P *= 0.72):** I1: 2.9 ± 2 days I2: 3.1 ± 2.3 days	I1: 5 ± 1.5 I2: 5 ± 1.5
	Lundstrom, 2007 [35]	DSM-IV, OBS-scale	POD 1-discharge	Orthopedic	Comprehensive intervention (staff education, team work, individual care planning, prevention and treatment delirium and delirium-related complications) *vs. *standard	I1: 74/28 I2: 74/23	I1: 82 ± 7 I2: 82 ± 6	9	I1: 56/102 (54.9%) I2: 73/97 (75.3%)	0.003	**Duration (*P *= 0.009):** I1: 5.0 ± 7.1 days I2: 10.2 ± 13.3 days	I1: 28 ± 17.9 I2: 38 ± 40.6

**H_2 _receptor blockers**	Kim, 1996 [21]	DSM-III	POD1, discharge	Cardiovascular	Cimetidine *vs. *ranitidine	I1: 14/39 I2: 17/41	I1: 68 ± 10 I2: 64 ± 11	4	I1: 13/53 (24.5%) I2: 15/58 (25.9%)	0.872		I1: 8.9 ± 3.9 I2: 8.7 ± 2.9

N/A, not available; POD, postoperative day; pre-, preoperative day; DDS, Delirium Detection Score; DSM, Diagnostic and Statistical Manual of Mental Disorders; CAM, Confusion Assessment Method; OBS, organic brain syndrome; DRS, Delirium Rating Scale; DSI, Delirium Symptom Interview; MDAS, Memorial Delirium Assessment Scale; ICDSC, Intensive Care Delirium Screening Checklist; sl, sublingually; po, orally; im, intramuscularly; iv, intravenously; bid, *bis in die*; tid, *ter in die*.

### Quality scores of included studies

The scores of included studies were shown in Table 4. The scores ranged from 3 [18] to 12 [36,52,54]. The average score was 8.3 with a standard deviation of 2.2. A score lower than 6 was found in four studies [17,18,21,22]. Three studies got the full score 12 [36,52,54].

**Table 4:** Methodological quality scores of included trial reports

Study	Randomization	Allocation concealment	Blinding	Withdrawal or dropouts	ITT analysis	Delirium assessor blinding	Baseline similarity	Delirium follow-up	Total
Williams-Russo, 1999 [23]	2	2	0	1	1	1	1	2	10
Berggren, 1987 [17]	1	1	0	1	1	1	0	0	5
Williams-Russo, 1995 [20]	2	1	0	1	1	1	1	2	9
Kudoh, 2004 [27]	2	1	0	1	1	1	0	2	8
Papaioannou, 2005 [31]	2	1	0	1	1	0	1	1	7
Sieber, 2010 [47]	2	1	0	1	1	1	1	2	9
Nishikawa, 2004 [28]	1	2	0	1	1	1	0	1	7
Hudetz, 2009 [40]	1	2	0	1	0	1	1	2	8
Royse, 2011 [51]	2	2	1	1	0	1	1	0	8
Leung, 2006 [34]	2	2	0	0	0	1	1	0	6
Williams-Russo, 1992 [19]	2	1	0	1	0	1	0	2	7
Mann, 2000 [24]	2	1	0	1	0	1	1	2	8
Beaussier, 2006 [32]	2	1	2	1	0	1	0	2	9
Mouzopoulos, 2009 [43]	2	1	0	1	0	0	1	2	7
Musclow, 2012 [53]	2	2	2	1	1	1	0	2	11
Maldonado, 2009 [41]	2	1	0	1	1	1	1	1	8
Shehabi, 2009 [44]	2	1	2	1	1	1	1	2	11
Rubino, 2010 [46]	1	2	2	1	1	1	0	0	8
Liptzin, 2005 [30]	1	1	2	1	0	1	0	0	6
Sampson, 2007 [37]	2	2	2	1	0	1	0	2	10
Marcantonio, 2011 [48]	2	1	2	1	0	1	0	2	9
Gamberini, 2009 [39]	2	1	2	1	0	1	1	2	10
Kaneko, 1999 [22]	1	2	0	1	0	0	1	0	5
Kalisvaart, 2005 [29]	2	2	2	1	1	1	1	1	11
Wang, 2012 [54]	2	2	2	1	1	1	1	2	12
Larsen, 2010 [45]	1	1	2	1	1	1	0	2	9
Prakanrattana, 2007 [36]	2	2	2	1	1	1	1	2	12
Hakim, 2012 [52]	2	2	2	1	1	1	1	2	12
Leung, 2006 [33]	2	1	2	1	1	1	1	1	10
Pesonen, 2011 [50]	2	1	2	1	0	1	1	2	10
Aizawa, 2002 [26]	1	1	0	1	1	1	1	2	8
Taguchi, 2007 [38]	2	1	0	1	1	0	0	2	7
Ono, 2011 [49]	2	1	0	1	0	0	0	2	6
Schindler, 1989 [18]	1	1	0	1	0	0	0	0	3
McCaffrey, 2009 [42]	2	1	0	1	1	0	0	1	6
Marcantonio, 2001 [25]	2	2	0	1	1	1	0	2	9
Lundstrom, 2007 [35]	2	2	0	1	1	1	0	2	9
Kim, 1996 [21]	1	1	0	1	0	1	0	0	4

### Quantitative review and meta-analysis

#### Category 1. Perioperative procedures and drugs (Table 2)

##### 1.1 Controlled hypotension

Williams-Russo *et al. *[23] tested the effects of induced hypotension by epidural anesthesia on delirium in patients accepting hip replacement surgery. Intraoperative mean arterial blood pressure (MAP) was maintained in the range of 45 to 55 (*n *= 117) or 55 to 70 mmHg (*n *= 118). They found no difference in the incidences of postoperative delirium (8.5% *vs. *4.2%; MAP 45 to 55 *vs. *MAP 55 to 70, *P *= 0.167). Power calculations suggested that 675 patients per group would be needed to observe a significant difference in delirium occurrence based on the reported incidences but this study included a total of 235 patients.

##### 1.2 Neuraxial anesthesia versus general anesthesia

**Figure 2:**
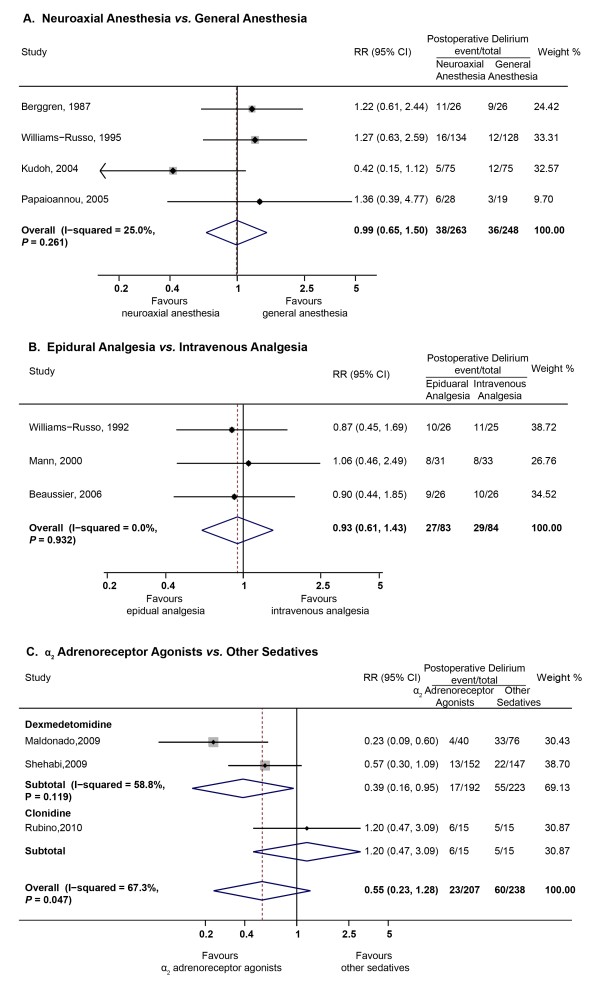
**Summary relative risks (RRs) for the incidences of postoperative delirium in trials comparing different anesthesia (A), analgesia (B) and postoperative sedation (C) methods**.

We identified four studies with 511 patients [17,20,27,31] that compared the effects of different anesthesia methods on postoperative delirium. Meta-analysis using a fixed-effects model (χ^2^_(3) _= 4, *P *= 0.261, I^2 ^= 25%) revealed no difference between neuraxial and general anesthesia (pooled RR = 0.99, 95% CI = 0.65 to 1.50, *P *= 0.962, Figure 2A). The pooled incidences based on a random-effects model were 17.1% (95% CI = 7.8% to 37.8%) for neuraxial anesthesia and 17.1% (95% CI = 9.3% to 31.4%) for general anesthesia.

##### 1.3 Sedation depth during spinal anesthesia

Sieber *et al. *[47] tested whether patients receiving deep sedation during spinal anesthesia would suffer from more postoperative delirium. Bispectral index (BIS) was kept at approximately 50 in the deep sedation group (*n *= 57) and at 80 or higher in the light sedation group (*n *= 57) during surgery. The study showed an increased incidence of postoperative delirium (40.4% *vs. *19.3%; deep sedation *vs. *light sedation, *P *= 0.014) and a significant longer duration of delirium (1.4 ± 4.0 *vs. *0.5 ± 1.5 days; deep sedation *vs. *light sedation, *P *= 0.01) in the deep-sedated patients.

##### 1.4 General anesthetics

Patients receiving propofol for general anesthesia showed higher Delirium Rating Scale (DRS) scores compared to patients receiving sevoflurane (6 ± 3 *vs. *2 ± 1, *P *= 0.002) in the study by Nishikawa *et al. *[28]. There was no difference in the incidences of postoperative delirium (16% *vs. *0, propofol *vs. *sevoflurane, *P *= 0.110). Power calculations suggested that 58 patients per group would be needed to achieve a significant difference in the incidences of delirium but the current study only included 25 patients per group.

Royse *et al. *[51] tested the influence of either propofol or desflurane on the incidence of postoperative delirium in patients undergoing coronary artery bypass surgery. Seven of 89 patients receiving propofol and 12 of 92 patients receiving desflurane developed delirium. No difference was found in incidence between the two groups (7.9% *vs. *13.2%, propofol *vs. *desflurane, *P *= 0.245). Power calculations suggested that 732 patients per group would be needed to observe a significant difference in delirium occurrence based on the reported incidences but a total of 171 patients were enrolled in the study.

Less postoperative delirium was found in patients receiving additional ketamine (0.5 mg/kg intravenously, single bolus) for anesthesia induction compared to standard methods (3.45% *vs. *31.03%; ketamine *vs. *standard, *P *= 0.012). Hudetz *et al. *[40] recruited 29 patients undergoing cardiac surgery per group for this study.

Leung *et al. *[34] found no additional effect of N_2_O on the development of postoperative delirium compared to standard anesthesia in older patients undergoing noncardiac surgery. Forty-four of 105 patients (41.9%) exposed to additional N_2_O and 46 of 105 patients (43.8%) receiving standard anesthesia developed delirium. Power calculations suggested that 14,524 patients per group would be needed to get a difference in delirium occurrence based on the reported incidences.

##### 1.5 Epidural analgesia versus intravenous analgesia

Three RCTs with 167 patients [19,24,32] tested whether epidural analgesia was superior to intravenous analgesia in preventing postoperative delirium in older patients undergoing major orthopedic and abdominal surgeries. Meta-analysis using a fixed-effects model (χ^2^_(2) _= 0.14, *P *= 0.932, I^2 ^= 0) found no difference between epidural and intravenous analgesia (pooled RR = 0.93, 95% CI = 0.61 to 1.43, *P *= 0.751, Figure 2B). The pooled incidences utilizing a fixed-effects model were 33.4% (95% CI = 23.8% to 47.0%) for epidural analgesia and 36.7% (95% CI = 26.6% to 50.4%) for intravenous analgesia. Power calculations suggested that 4,391 patients per group would be needed to observe a significant difference in delirium occurrence based on the pooled incidences but a total of 167 patients were recruited in the identified three trials.

##### 1.6 Additional fascia iliaca compartment block

Mouzopoulos *et al. *[43] investigated the effects of additional fascia iliaca compartment block (FICB, 0.25% bupivacaine 0.3 mL/kg) on postoperative delirium in hip surgery patients who were at intermediate or high risk for delirium. Patients included had to have at least one of the four predictive risk factors (severity of illness, cognitive impairment, index of dehydration and visual impairment) as described by Inouye *et al. *[55,56]. There were 102 patients receiving additional FICB plus standard analgesia and 105 patients receiving standard analgesia only. The FICB prophylaxis group showed decreased incidence (10.8% *vs. *23.8%; additional FICB *vs. *standard, *P *< 0.001), reduced severity (DRS scale, 14.34 ± 3.6 *vs. *18.61 ± 3.4; additional FICB *vs. *standard, *P *< 0.001) and shortened duration of delirium (5.22 ± 4.28 *vs. *10.97 ± 7.16 days, additional FICB *vs. *standard, *P *< 0.001). The study was accompanied with insufficient allocation concealment, blinding and no intention-to-treat (ITT) analysis.

##### 1.7 Long-acting morphine

Musclow *et al. *[53] reported increased severity of postoperative delirium using the NEECHAM scale in patients receiving long-acting morphine (28.70 ± 1.82 *vs. *29.14 ± 0.61; morphine *vs. *placebo, *P = *0.02) which was administered at an oral dose of 30 mg, twice daily for three days. There was no difference in the incidences of delirium (10.3% *vs. *3.4%; morphine *vs. *placebo, *P = *0.082). Power calculations suggested that 524 patients would be needed to observe a difference in delirium occurrence based on the reported incidences but this study enrolled 190 patients.

##### 1.8 Postoperative sedation using alpha-2 adrenoreceptor agonists

Three RCTs with 445 patients [41,44,46] tested whether alpha-2 adrenoreceptor agonists (dexmedetomidine and clonidine) were superior to other sedatives in preventing postoperative delirium in patients undergoing cardiovascular surgery. Meta-analysis using a random-effects model (χ^2^_(2) _= 5.71, *P *= 0.057, I^2 ^= 65) found no difference between alpha-2 adrenoreceptor agonists and other sedatives (pooled RR = 0.55, 95% CI = 0.23 to 1.28, *P *= 0.163, Figure 2C). The pooled incidences utilizing a fixed-effects model were 15.2% (95% CI = 5.3% to 43.6%) for alpha-2 adrenoreceptor agonists and 25.1% (95% CI = 10.1% to 62.1%) for other sedatives. Power calculations suggested that 686 patients would be needed to get a difference in delirium occurrence based on the pooled incidences but a total of 445 patients were included. Subgroup analysis found that dexmedetomidine was more effective than other sedatives in preventing postoperative delirium (pooled RR = 0.39, 95% CI = 0.16 to 0.95, *P *= 0.039). Besides the effects on the incidences of delirium, Maldonado *et al. *[41] found that dexmedetomidine (loading dose: 0.4 μg/kg, maintenance drip of 0.2 to 0.7 μg/kg/hour) had no effect on the duration of delirium in patients with delirium (2.0 ± 0 *vs. *3.0 ± 3.1 *vs. *5.4 ± 6.6 days; dexmedetomidine *vs. *propofol *vs. *midazolam, *P *= 0.82). Shehabi *et al. *[44] found dexmedetomidine (0.1 to 0.7 μg/kg/hour) was superior to propofol in shortening the duration of delirium (2 ± 4 *vs. *5 ± 8 days; dexmedetomidine *vs. *propofol, *P *= 0.032). Rubino *et al. *[46] found supplemental clonidine (loading dose: 0.5 μg/kg, maintenance drip of 1 to 2 μg/kg/hour) was able to reduce the severity of delirium (DDS, 0.6 ± 0.7 *vs. *1.8 ± 0.8, additional clonidine *vs. *standard, *P *< 0.001).

#### *Category 2*. Pharmacological, psychological or multicomponent interventions (Table 3)

##### 2.1 Acetylcholinesterase inhibitors

**Figure 3:**
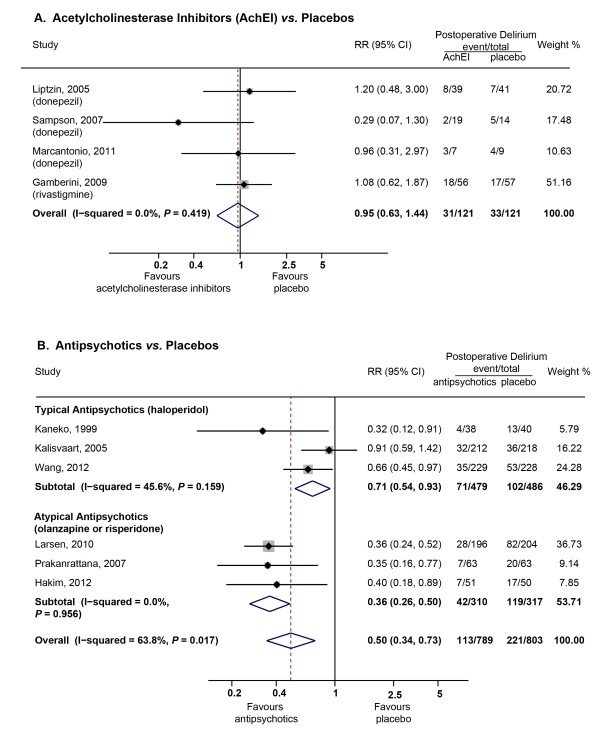
**Summary relative risks (RRs) for the incidences of postoperative delirium in trials testing the role of acetylcholinesterase inhibitors (AchEI) (A) and antipyschotics (B)**.

Four RCTs with 242 patients tested whether elevating brain acetylcholine levels by acetylcholinesterase inhibitors (AchEI) would be helpful for preventing postoperative delirium in patients accepting major orthopedic surgeries [30,37,39,48]. Three studies used oral donepezil (5 mg/day, 4 to 30 days) [30,37,48] and one study used oral rivastigmine (4.5 mg/day, 7 days) [39]. Meta-analysis using fixed-effects model (χ^2^_(3) _= 2.83, *P *= 0.419, I^2 ^= 0) found no difference between the two groups on the incidences of postoperative delirium (pooled RR = 0.95, 95% CI = 0.63 to 1.44, *P *= 0.825, Figure 3A). The pooled incidences utilizing fixed-effects model were 28.5% (95% CI = 20.6% to 39.5%) for patients taking acetylcholinesterase inhibitors and 36.1% (95% CI = 26.7% to 48.7%) for patients taking placebos. Power calculations suggested that 794 patients per group would be needed to observe a significant difference in delirium occurrence based on the pooled incidences and 121 patients per group in the existing four studies were included. Besides no effects on the incidences of delirium, Liptzin *et al. *[30] found that donepezil failed to shorten the duration of delirium (1 ± 0 *vs. *1.3 ± 1.2 days, donepezil *vs. *placebo, *P *= 0.12). Marcantonio *et al. *[48] reported that donepezil did not reduce the severity of delirium (Memorial Delirium Assessment Scale (MDAS) changes,1.3 ± 2.5 *vs. *1.6 ± 5.2, donepezil *vs. *placebo, *P *= 0.91) but only 16 patients were included in the study.

##### 2.2 Antipsychotics

We identified six trials with 1,592 patients which tested the role of antipsychotics on preventing postoperative delirium. Three trials used the typical antipsychotic haloperidol [22,29,54]. The doses varied from 1.5 mg/day to 5 mg/day with a duration of one to five days. The other three trials used atypical antipsychotics risperidone and olanzapine [36,45,52]. Risperidone was given sublingually once after surgery at a dose of 1 mg in the study by Prakanrattana *et al. *[36]. Hakim *et al. *[52] recruited patients with subsyndromal delirium only and risperidone was continually given orally at a dose of 1 mg/day until 24 hours after subsidence of subsyndromal delirium or a score of more than 3 on the Intensive Care Delirium Screening Checklist (ICDSC) was obtained. Oral olanzapine was given at a dose of 5 mg just before and after surgery in the study by Larsen *et al. *[45]. Meta-analysis using a random-effects model (χ^2^_(5) _= 13.82, *P *= 0.017, I^2 ^= 63.8) found a significant difference between antipsychotics and placebo (pooled RR = 0.50, 95% CI = 0.34 to 0.73, *P *= 0.000, Figure 3B). Meta-regression showed that the heterogeneity came from the kind of antipsychotics used (typical or atypical; REML estimate of between-study variance = 0, proportion of between-study variance explained = 100%). Subgroup analysis suggested that both typical and atypical antipsychotics were able to prevent postoperative delirium (RR = 0.71, 95% CI = 0.54 to 0.93 for typical antipsychotics and RR = 0.36, 95% CI = 0.26 to 0.50 for atypical antipsychotics). Indirect comparison using ITC tools [57] found a superior role of atypical antipsychotics in preventing delirium compared to typical antipsychotics (estimated RR = 1.95, 95% CI = 1.28 to 2.96, *P *= 0.072). The pooled incidences utilizing a fixed-effects model were 14.5% (95% CI = 12.1% to 17.3%) for patients receiving antipsychotics and 28.4% (95% CI = 21.0% to 38.5%) for patients taking placebo. Besides the effects on the incidences of delirium, Kalisvaart *et al. *[29] found that haloperidol reduced the severity (DRS, 14.40 ± 3.5 *vs. *18.41 ± 4.4, haloperidol *vs. *placebo, *P *< 0.001) and shortened the duration of delirium (5.41 ± 4.91 *vs. *11.85 ± 7.56 days, haloperidol *vs. *placebo, *P *< 0.001) in patients suffering from delirium. Larsen *et al. *[45] reported that olanzapine increased the severity (DRS), 16.44 ± 3.7 *vs. *14.5 ± 2.7, haloperidol *vs. *placebo, *P *= 0.02) and duration of delirium (2.2 ± 1.3 *vs. *1.6 ± 0.7 days, haloperidol *vs. *placebo, *P *= 0.02). Hakim *et al. *[52] found that risperidone had no effect on the duration of delirium in patients with postoperative delirium (3 ± 1.5 *vs. *3 ± 0.8 days, risperidone *vs. *placebo, *P *= 0.669).

##### 2.3 Anticonvulsants

Leung *et al. *[33] tested whether oral gabapentin (900 mg/day, for four days) was helpful in preventing postoperative delirium in older patients undergoing spine surgery. Delirium was identified in none of the nine patients receiving gabapentin and in five of the twelve patients receiving placebo (*P *= 0.045). Pesonen *et al. *[50] randomly assigned oral pregabalin (150 mg/day, for six days, *n *= 35) or placebo (*n *= 35) to patients accepting cardiac surgery. Their study found that pregabalin was able to reduce the severity of delirium (CAM-ICU, 24 ± 8 *vs. *21 ± 19, *P *= 0.04).

##### 2.4 Sleep restoration using diazepam, flunitrazepam and pethidine

Aizawa *et al. *[26] tested whether restoring sleep-wake cycle with medications after surgery was useful to prevent postoperative delirium. The researchers randomly divided 40 patients accepting major abdominal surgeries into two groups. The experimental group (*n *= 20) received standard treatment plus diazepam/flunitrazepam/pethidine (DFP) for three days to improve sleep disorders and the control group underwent standard treatment (*n *= 20). Less delirium was developed in the DFP group (5% *vs. *35%, DFP *vs. *standard, *P *= 0.023).

##### 2.5 Sleep restoration using bright light

We identified two studies [38,49] with 33 patients that tested the hypothesis that improving the sleep-wake cycle using bright light (two hours per day in the morning, 2500 to 5000 lx) would be useful to prevent delirium. Meta-analysis using a fixed-effects model (χ^2^_(1) _= 0.15, *P *= 0.703, I^2 ^= 0) found no difference between bright light and control (pooled RR = 0.30, 95% CI = 0.07 to 1.26, *P *= 0.099). The pooled incidence utilizing a fixed-effects model were 13.0% (95% CI = 2.1% to 78.8%) for bright light and 41.3% (95% CI = 20.9% to 81.5%) for control. Power calculations suggested that 50 patients per group would be needed to get a significant difference in delirium occurrence based on the pooled incidences but only a total of 33 patients were included in the two trials. Besides the effects on the incidences of delirium, Taguchi *et al. *[38] found bright light therapy reduced the severity of delirium (NEECHAM, 6.7 ± 0.7 *vs. *21.1 ± 7, bright light *vs. *standard, *P *= 0.014).

##### 2.6 Psychological intervention

Schindler *et al. *[18] detected the role of active daily psychological intervention on postoperative delirium in patients undergoing cardiac surgery. No difference was found on the incidences of delirium between the two groups (0 *vs. *12.5%; psychological intervention *vs. *standard, *P *= 0.227). Power calculations suggested that a total of 154 patients were needed to observe a difference in delirium occurrence based on the reported incidences but the study included only 33 patients.

##### 2.7 Music

McCaffrey *et al. *[42] recruited 22 patients (11 patients per group) and evaluated the effects of music on delirium prevention. The patients in the music group received standard hospital care plus listening to soothing lullaby music at least four times a day for one hour. Delirium severity was detected using NEECHAM confusion scale on each of the first three postoperative days. They found that listening to music decreased the severity of delirium (NEECHAM, 24 ± 0.97 *vs. *22.5 ± 1.22, music *vs. *standard, *P *= 0.000).

##### 2.8 Multicomponent interventions

Multicomponent interventions that combined both pharmacological and non-pharmacological strategies were performed to prevent postoperative delirium in two RCTs with 325 patients [25,35]. Meta-analysis using a fixed-effects model (χ^2^_(1) _= 0.55, *P *= 0.608, I^2 ^= 0) found that multicomponent interventions decreased the incidence of postoperative delirium (pooled RR = 0.71, 95% CI = 0.58 to 0.86, *P *= 0.000). The pooled incidences were 43.3% for multicomponent interventions and 62.4% for standard treatment. In addition, Marcantonio *et al. *[25] found multicomponent interventions reduced the number of patients with severe delirium (7/62 (11.3%) *vs. *18/64 (28.1%); multicomponent interventions *vs. *standard, *P *= 0.02) and had no effect on the duration of delirium in patients suffering from delirium (2.9 ± 2 *vs. *3.1 ± 2.3 days; multicomponent interventions *vs. *standard, *P *= 0.73). Lundstrom *et al. *[35] reported that multicomponent interventions shortened the duration of delirium (5.0 ± 7.1 *vs. *10.2 ± 13.3 days; multicomponent interventions *vs. *standard, *P *= 0.009).

##### 2.9 Histamine H_2 _receptor blockers

Kim *et al. *[21] found no difference in the incidences of postoperative delirium between cimetidine and ranitidine in postoperative cardiac surgical patients. The incidences were close (24.5% *vs. *25.9%, cimetidine *vs. *ranitidine, *P *= 0.872). More than 20,000 patients for each group would be needed to observe a significant difference in the incidences of delirium and a total of 111 patients were included in the study.

#### Interventions effective in preventing postoperative delirium did not shorten the length of hospital stay

**Figure 4:**
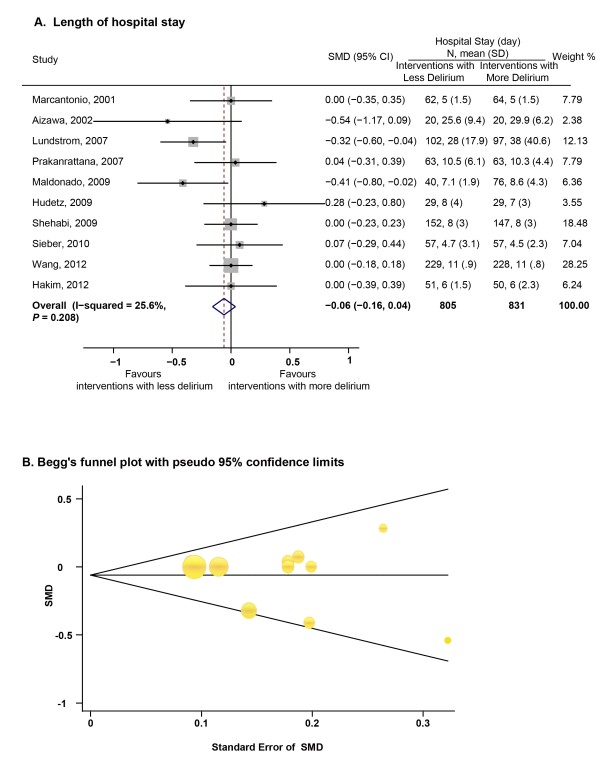
**Interventions successful in preventing postoperative delirium failed to shorten the length of hospital stay**. **(A) **Summary standard mean differences (SMDs) for the length of hospital stay between interventions with less delirium and interventions with more delirium. **(B) **Begg's funnel plot with effect measures (SMD) as a function of its standard error (SE) for the length of hospital stay.

We identified 10 studies with 1,636 patients reporting both different incidences of postoperative delirium between the two interventions and inpatient time [25,26,35,36,40,41,44,47,52,54]. Meta-analysis using a fixed-effects model (χ^2^_(9) _= 12.1, *P *= 0.208, I^2 ^= 25.6%) found no significant difference in the length of hospital stay between interventions with lower or higher incidences of postoperative delirium (pooled SMD = -0.06, 95% CI = -0.16 to 0.04, *P *= 0.159, Figure 4A). The pooled incidences based on the fixed-effects model were 16.1% for interventions with less delirium and 35.4% for interventions with more delirium. No significant publication bias was found by Begg's test (z = 0.54, P > |z| = 0.592) and by visual inspection of the funnel plot (Figure 4B).

## Discussion

### Summary of evidence

The main findings to emerge from this systematic review and meta-analysis include: 1) There was a huge heterogeneity of interventions among trials. Most of the interventions suffered from only a handful of studies and small sample sizes. Some studies suffered from methodological defects and were at high risk of bias [17,18,21,22]. These shortcomings disallowed sufficient interpretation of the effectiveness of interventions. 2) The meta-analysis showed that dexmedetomidine sedation, multicomponent interventions and antipsychotics were useful in preventing postoperative delirium. 3) Based on the result and quality of individual study, it appeared that light sedation during spinal anesthesia, additional ketamine during anesthesia induction, additional fascia iliaca compartment block, anticonvulsants and sleep restoration using diazepam/flunitrazepam/pethidine were useful in preventing postoperative delirium. 4) The meta-analysis found that interventions useful for preventing postoperative delirium did not shorten the patients' time spent in the hospital. Table 5 provides a summary of the efficacy of interventions for diminishing delirium.

**Table 5:** Summary of the efficacy of the interventions.

Conclusions based on	Perioperative procedures and drugs (>, superior to; =, equally effective to; UC*, uncertain)	Pharmacological, psychological or multicomponent interventions (>, superior to; =, equally effective to; *UC*, uncertain)
**Meta-analysis**	Postoperative sedation: dexmedetomidine > other sedatives [41,44]	Typical antipsychotics > placebo [22,29,54] Atypical antipsychotics > placebo [36,45,52] Multicomponent interventions > standard [25,35]
	Anesthesia type: neuraxial = general [17,20,27,31] Analgesia type: epidural = intravenous [19,24,32]	Acetylcholinesterase inhibitors *= *placebo [30,37,39,48]
		Atypical antipsychotics *UC *typical antipsychotics [22,29,36,45,52,54] Sleep restoration: bright light *UC *standard [38,49]

**Single study**	Sedation depth: light > deep [47] Additional ketamine > routine general anesthesia induction [40] Additional fascia iliaca compartment block > standard analgesia [43]	Anticonvulsants: gabapentin > placebo [33]; pregabalin > placebo [50] Sleep restoration: diazepam/flunitrazepam/pethidine > standard [26]
	Additional N_2_O during general anesthesia = standard [34]	Histamine H_2_ blockers: cimetidine *UC *ranitidine [21] Psychiatric intervention *UC *standard [18] Music *UC *standard [42]
	Controlled hypotension: mild *UC *marked [23] Anesthetics plus epidural anesthesia: propofol *UC *sevoflurane [28] Anesthetics during general anesthesia: propofol *UC *desflurane [51] Analgesics: Long-acting morphine *UC *placebo [53] Postoperative sedation: clonidine *UC *other sedatives [46]	

*The efficacy is considered uncertain if there is more than 50% difference in incidences of delirium between the two interventions, or if the quality score of the trial is below 6.

Consistent with our results, Lin *et al. *[58,59] found that dexmedetomidine sedation was inversely related with the incidence of delirium in patients with cardiac surgery. Contrarily, Tan *et al. *[60] concluded that the use of dexmedetomidine in critically ill adult patients had no effect on delirium in their meta-analysis. Besides the different doses and durations of dexmedetomidine use, patients with different illnesses might develop delirium due to different reasons [61], which might help explain the discrepancy.

Lonergan *et al. *[62] found that both typical antipsychotics (haloperidol) and atypical antipsychotics (olanzapine, risperidone and quetiapine) were effective in treating delirium in their meta-analysis. These results were consistent with the current meta-analysis which tested the role of antipsychotics on delirium prevention. Studies by Devlin *et al. *[63,64] and Skrobik *et al. *[65] also found a positive role of quetiapine and olanzapine in treating delirium in critically ill patients. Campbell *et al. *[66] found no superiority for second-generation antipsychotics over haloperidol in managing delirium. Devlin *et al. *[67] had critically reviewed six studies [54,65,68-71] which used haloperidol to prevent or treat delirium in noncritically or critically ill patients. Only the study by Wang *et al. *[54] showed that low-dose haloperidol reduced the incidence of delirium compared to placebo. The inconsistent results of haloperidol might be due to the following reasons: 1) There was a great heterogeneity of the patient populations among the six studies [54,65,68-71]. These studies included patients with severe illnesses (AIDS and cancer) and patients accepting different surgeries or critically ill patients. 2) The comparator of haloperidol was different among studies. Girard *el al. *[71] used both atypical antipsychotics and placebo as the control. Wang *et al. *[54] used placebo as the control. Chlorpromazine and lorazepam were used as the control for haloperidol in the study by Breitbart *et al. *[68]. Another three studies [65,69,70] used atypical antipsychotics (olanzapine or risperidone or ziprasidone) as the control. 3) The dosage and duration of haloperidol differed greatly among studies.

In support of the National Institute for Health and Clinical Excellence guidelines recommending an individual multicomponent intervention package aiming to prevent delirium [72,73], two studies included in our meta-analysis supported multicomponent interventions as a useful way to prevent postoperative delirium [25,35]. A recent study also showed a 30% reduction of delirium by multimodal geriatric consultation versus usual care in older adults with recent hip fracture [74]. Additional similar studies are being performed, which might add evidence to the finding [75-77].

Our meta-analysis data found no difference in the length of patient hospital stay between interventions with higher incidences of delirium (pooled incidence, 35.4%) and interventions with lower incidences (pooled incidence,16.1%). This finding was contrary to results from previous observational studies which showed that patients with postoperative delirium stayed longer in the hospital [4,78-80]. Resolving these differences is important as prolonged hospital stay is a heavy burden on the health care system [5] and should also be included as an important clinical outcome during delirium prevention [67]. However, as only 21 of the 38 included trials reported the inpatient time, there was a potential publication bias. Furthermore, our meta-analysis included heterogeneous studies with huge differences in both the incidences of postoperative delirium and the time of hospital stay. Further clinical trials with homogenous patients receiving similar interventions might help to clarify this issue.

### Limitations

This systematic review and meta-analysis had several limitations. 1) We included different types of surgeries for a single intervention and this possibly affected the heterogeneity. 2) Multiple methods and different frequencies of postoperative delirium screening across the studies were another source of heterogeneity [1]. 3) For the same intervention, the dose and duration varied greatly among studies and this might account for different effects [70]. 4) Application of the scoring system developed for this study revealed methodological defects in some studies. These defects added a degree of uncertainty to the present results. For example, given that delirium is a multifactorial disorder, similar baseline data were essential when comparing the effects of two interventions. However, 18 [17-19,21,25,27,28,30,32,35,37,38,42,45,46,48,49,53] of the 38 studies did not adjust the risk factors before grouping. 5) Publication bias might account for some of the effects reported here. Most of the included studies were small-sampled single-centered studies with less methodological rigor than large-sampled studies. This factor might contribute to an overestimation of effect sizes in small trials. 6) We excluded homogeneous populations of patients with dementia in our study [81,82]. In addition, only two studies [25,35] stated that they included a small subpopulation of patients with dementia. Considering the high morbidity of dementia in the adults and the overlap of dementia with, and contribution to, delirium [3,83-85], we have excluded a large group of patients who were susceptible to postoperative delirium. This exclusion should be seen as a source of potential selection bias and could limit the interpretation of our findings.

### Future directions

Our review raised several questions that need to be addressed in future studies: 1) There were three types of postoperative delirium: hyperactive (25%), hypoactive (50%) and mixed (25%) delirium which had different causes and consequences [1,16,86]. However, none of the existing studies tried to distinguish them or tested the specific effects of interventions. Future studies should include screening tools such as the Richmond Agitation-Sedation Scale (RASS) to classify the subtypes of delirium [87] and test their reactions to various interventions. 2) The severity and duration of delirium needed more attention. Moreover, the severity and duration of delirium should be averaged for all patients but not only for patients with delirium. 3) High-risk and low-risk patients might show different sensitivity to precipitating factors and interventions. Thus, there is a need for future studies that stratify high-risk patients and low-risk patients in delirium assessment. We identified only two studies stratifying the risk of delirium [29,43]. One study only included patients with subsyndromal delirium [52]. Further studies using valid risk-stratifying tools for delirium [12] can make a contribution to this important clinical problem.

## Conclusions

Heterogeneity and small sample sizes precluded conclusions regarding the interventions that are likely to prevent postoperative delirium. The limited data suggested that the efficacious way to prevent postoperative delirium included dexmedetomidine sedation, multicomponent interventions and antipsychotics comprising haloperidol, olanzapine and risperidone. Anesthesia types and analgesia methods had no bearing on delirium. Acetylcholinesterase inhibitors were ineffective in preventing delirium. Interventions effective in preventing postoperative delirium did not shorten the length of hospital stay. Considered together, these findings suggested an urgent need for high-quality large-scale RCTs.

## Key messages

• Multiple strategies including perioperative management procedures, pharmacological and nonpharmacological interventions have been used in an effort to prevent postoperative delirium.

• There is a consensus in the data that dexmedetomidine sedation, multicomponent interventions and antipsychotics are useful in preventing postoperative delirium.

• Anesthesia types and analgesia methods have no bearing on postoperative delirium.

• Acetylcholinesterase inhibitors are ineffective in preventing postoperative delirium.

• Reduced postoperative delirium is not related with shortened hospital stay.

## Abbreviations

AchEI: acetylcholinesterase inhibitors; BIS: bispectral index; CAM: Confusion Assessment Method; CI: confidence interval; DDS: Delirium Detection Score; DFP: diazepam/flunitrazepam/pethidine; DRS: Delirium Rating Scale; DSI: Delirium Symptom Interview; DSM: Diagnostic and Statistical Manual of Mental Disorders; FICB: fascia iliaca compartment block; ICD: International Statistical Classification of Diseases and Related Health Problems; ICDSC: Intensive Care Delirium Screening Checklist; ICU: intensive care unit; ITT: intention-to-treat; MAP: mean arterial blood pressure; MDAS: Memorial Delirium Assessment Scale; N/A: not available; NS: not significant; OBS: organic brain syndrome; POD: postoperative day; pre-: preoperative day; RASS: Richmond Agitation-Sedation Scale: RCT: randomized controlled trial; RR: risk ratio; SD: standard definition; SE: standard error; SMD: standard mean difference.

## Competing interests

Dr. Xue-Yin Shi receives grants from the National Natural Science Foundation of China (No. 81070880), the 12th Five-Year Key Project of PLA (No. BWS12J027) and Key Basic Research Projects of the Science and Technology Commission of Shanghai Municipality, China (No. 12JC1410902). The three grants financed this article. Hao Zhang, Yan Lu, Meng Liu, Zui Zou, Long Wang and Feng-Ying Xu declared no competing interests.

## Authors' contributions

HZ, YL and XS conceived and designed the study. HZ and YL carried out the literature search. ML, ZZ and XS carried out the data extraction. FX, LW and XS carried out the quality assessment. HZ and YL analyzed and interpreted the data. HZ, YL and XS prepared and revised the manuscript. All authors have read and approved the manuscript.

## Supplementary Material

Additional file 1 **PRISMA Checklist**. This file contains a table of the PRISMA 2009 Checklist in which we checked and noted what had been done according to the guidelines of the PRISMA statement for the current systematic review and meta-analysis. Click here for file
